# The greening reaction of skipjack tuna (*Katsuwonus pelamis*) metmyoglobin promoted by free cysteine during thermal treatment

**DOI:** 10.7717/peerj.13923

**Published:** 2022-08-17

**Authors:** Andrés Álvarez-Armenta, Ramón Pacheco-Aguilar, Alonso A. López-Zavala, David O. Corona-Martínez, Rogerio R. Sotelo-Mundo, Karina D. García-Orozco, Juan C. Ramírez-Suárez

**Affiliations:** 1 Laboratorio de Bioquímica y Calidad de Productos Pesqueros, Tecnología de Alimentos de Origen Animal, Centro de Investigación en Alimentación y Desarrollo A. C., Hermosillo, Sonora, México; 2 Departamento de Ciencias Químico Biológicas, Universidad de Sonora, Hermosillo, Sonora, Mexico; 3 Laboratorio de Estructura Molecular, Tecnología de Alimentos de Origen Animal, Centro de Investigación en Alimentación y Desarrollo, A. C., Hermosillo, Sonora, México

**Keywords:** Tuna greening, Sulfmyoglobin, Tuna myoglobin, Thermal treatment, Cysteine, Ferrylmyoglobin

## Abstract

**Background:**

Tuna muscle greening is a problem that occurs after heating. A hypothesis has been postulated to address this problem, involving a conserved Cys residue at position 10 (Cys-10) present on tuna myoglobin (Mb) that is exposed during the thermic treatment, forming a disulfide bond with free cysteine (Cys) in the presence of trimethylamine oxide (TMAO), resulting in the greening of the tuna Mb.

**Methods:**

We present a study using skipjack tuna (*Katsuwonus pelamis*) metmyoglobin (MbFe(III)-H_2_O) where the effect of free Cys (1–6 mM), TMAO (1.33 mM), and catalase on the greening reaction (GR) was monitored by UV-vis spectrometry during thermal treatment at 60 °C for 30 min. Moreover, the participation of Cys-10 on the GR was evaluated after its blocking with N-ethymaleimide.

**Results:**

The GR occurred in tuna MbFe(III)-H_2_O after heat treatment with free Cys, forming sulfmyoglobin (MbFe(II)-S) as the responsible pigment for the tuna greening. However, the rate constants of MbFe(II)-S production depended on Cys concentration (up to 4 mM) and occurred regardless of the TMAO presence. We postulate that two consecutive reactions involve an intermediate ferrylmyoglobin (promoted by H_2_O_2_) species with a subsequent MbFe(II)-S formation since the presence of catalase fosters the reduction of the rate reaction. Moreover, GR occurred even with blocked Cys-10 residues in tuna Mb and horse Mb (without Cys in its sequence).

**Discussion:**

We found that GR is not exclusive to tuna Mb´s, and it can be promoted in other muscle systems. Moreover, Cys and thermal treatment are indispensable for promoting this pigmentation anomaly.

## Introduction

One of the methods used to process and preserve muscle foods is heat treatment. This is used, among other beneficial effects, to reduce the bacterial content, provide notable organoleptic changes, and increase nutrient bioavailability (King & Whyte, 2006). The thermal treatment of meat products also accelerates protein and lipid oxidation due to increased pro-oxidant activity and iron release from the heme proteins (Kristensen & Andersen, 1997; Soladoye et al., 2015).

In the canning tuna industry, the greening of tuna muscle has been reported as an anomaly of discoloration by thermal treatment. However, although there is no data available related to the economic impact, considering that tuna greening is an unwanted muscle pigmentation, this results in financial loss for this industry (Singh, Mittal & Benjakul, 2022).

Grosjean et al. (1969) hypothesized this color change occurred during thermal treatment as the result of tuna metmyoglobin (MbFe(III)-H_2_O) interacting with free cysteine (Cys) (in the presence of trimethylamine oxide, TMAO), forming a disulfide bond between Cys and the only cysteine residue in the sequence (Cys-10) conserved in the tuna species. However, knowledge about this greening reaction (GR) is limited, and the mechanism remains uncertain.

Later, Padovani et al. (2016) proposed that free Cys in the presence of free iron from the heme group promotes the formation of superoxide anion (O_2_^•−^), H_2_O_2,_ and thiyl radicals (RS^•^). These oxidation products may lead to the formation of ferrylmyoglobin (MbFe(IV)=O) and later to the sulfmyoglobin (MbFe(II)-S). The green color is associated with the last molecule, which heme group of myoglobin is structurally modified to a chlorine type group, specifically the pyrrole B ring of porphyrin where a sulfur atom is added (Pietri, Román-Morales & López-Garriga, 2011; Libardi et al., 2014). However, Koizumi (1968) claims that species such as MbFe(II)-S, cholemyoglobin, and verdomyoglobin are pigments unrelated to the greening of tuna muscle. Therefore, we addressed the greening of the *Katsuwonus pelamis* tuna muscle during the thermal treatment.

## Materials and Methods

### Materials

Myoglobin from equine skeletal muscle, L-cysteine (Cys), trimethylamine oxide (TMAO), catalase from bovine liver, and N-ethylmaleimide (NEM) were purchased from Sigma Chemical Co. (St. Louis, MO, USA). In addition, skipjack tuna specimens (*Katsuwonus pelamis*) were taxonomically identified and frozen from a local provider. All other chemicals were of analytical grade.

### Extraction and purification of Mb from tuna muscle

Tuna was thawed (2–4 °C) and filleted, and the muscle was stored at –80 °C for further analysis. The extraction and purification of Mb from tuna muscle were conducted according to the following proposed methodology: Tuna muscle samples (50 g) were homogenized in a tissue homogenizer (Tizzumer SDT 1810; Tekmar Co., West Germany) with 150 mL of extraction buffer (50 mM Tris-HCl, pH 7.5; 0.1 mM PMSF; 1 mM EDTA; 25 g Triton X-100/L) at 13,000 rpm for 60 s. Then, the samples were centrifuged at 10,000 × *g* for 10 min at 4 °C (Avanti J-26S XPI; Beckman Coulter Inc., Palo Alto, CA, USA). The supernatant was recovered and filtered using Whatman #4 filter paper, and its pH was adjusted to 7.5. Next, the permeate was treated with ammonium sulfate fractionation at 65–100% saturation, agitated for 60 min, and then centrifuged at 30,000 × *g* for 20 min at 4 °C. Then, the precipitate obtained from the 100% saturation was dissolved with 10 mL of 50 mM Tris-HCl at pH 7.5 and dialyzed using a 10 kDa cut-off membrane (SnakeSkin dialysis tubing; Thermo Scientific, Waltham, MA, USA) against 20 volumes of the same buffer, with three buffer exchanges of 6 h each. To remove cytochrome *c* (Cyt *c*), which has similar spectral characteristics to those of Mb, the dialysate was injected into a cationic exchange column (HiTrap® SP HP, 5 mL), which was subsequently equilibrated with 50 mM Tris-HCl at pH 7.5 using an AKTA-FPLC chromatographer (GE Healthcare, Danderyd, Sweden). The no-retained fraction with absorbance at 280 and 410 nm was collected.

Afterward, the fractions with Mb were concentrated and subjected to size exclusion chromatography using a prepacked column (HiPrep 16/60 Sephacryl S-200 High Resolution), equilibrated with phosphate buffer (50 mM sodium phosphate, pH 7.5, and 150 mM NaCl). The elution was performed with the same buffer at a flow rate of 0.1 mL/min, collecting fractions of 2 mL that presented absorbance at 280 and 410 nm. Next, these fractions were pooled, concentrated, and subjected to a second size exclusion chromatography using a HiPrep 26/60 Sephacryl S-100 High-Resolution column with the same buffer but a flow of 0.2 mL/min. During the elution, the fractions (2 mL) were collected and analyzed by spectrophotometry (280 and 410 nm) and 17% SDS-PAGE (Laemmli, 1970) and stained with Coomassie Brilliant Blue R-250. SDS-PAGE was scanned using an automated gel-imaging instrument (Gel Doc EZ System; Bio-Rad Laboratories, Richmond, CA, USA). A broad-range-molecular-mass protein (Bio-Rad Laboratories, Richmond, CA, USA) and Mb from equine skeletal muscle (17 kDa) (Sigma-Aldrich, Saint Louis, MO, USA) were used as a standard.

### Preparation of metmyoglobin

The fractions of purified tuna Mb were pooled, and the protein content was adjusted to 0.1 mg/mL in phosphate buffer (0.1 M, pH 5.6, postmortem muscular pH). Next, the horse Mb was dissolved in the same buffer and filtered through syringe filters of regenerated cellulose (RC; 0.45 μm, Econofilter, Agilent, Santa Clara, CA, USA). Then, the MbFe(III)-H_2_O state (in both Mb´s) was obtained by oxidation with potassium ferricyanide (5 mg/mL) for 2 h at 25 °C (Tang, Faustman & Hoagland, 2004). Finally, the excess oxidant agent was removed by diafiltration using three buffer (0.1 M phosphate buffer, pH 5.6) exchanges using an Amicon Ultra-15 10K centrifugal filter. The Lowry method determined the protein concentration using bovine serum albumin as the protein standard (Lowry et al., 1951), and the final concentration was adjusted to 0.2 mg/mL before analysis.

### Blocking of Cys-10 in tuna Mb

To evaluate the role of Cys-10 on the GR, the reactive Cys-10 present in tuna Mb was blocked with N-ethylmaleimide (NEM) using the method reported by Helbo et al. (2014). Thus, the reaction was carried out in 50 mM phosphate buffer (pH 7) in a 3:1 (NEM: Mb) molar ratio for 1 h at room temperature. The excess NEM was removed by diafiltration using an Amicon Ultra-15 10K centrifugal filter with three buffer exchanges (0.1 M phosphate buffer, pH 5.6). The efficacy of the blocking was monitored by measuring thiol reactivity (conferred by Cys-10) with a 4,4-dithiodipyridine (4-PDS) assay (Grassetti & Murray, 1967). The reaction was carried out at a molar ratio of 4:1 (4-PDS: Mb) at room temperature, and the thiol-mediated conversion of 4-PDS to 4-thiopyridone was monitored at 324 nm.

### The effect of Cys and TMAO on the greening reaction

The effect of Cys and TMAO was tested in tuna MbFe(III)-H_2_O (plus Cys [4 mM] and/or TMAO [1.33 mM]) based on Grosjean et al. (1969). Absorption spectra were collected every 15 s during the thermic treatment (60 °C/30 min) in a quartz cuvette with 1 cm of optical length. A UV-vis Cary 50 (Varian, Inc. Agilent Technologies, Santa Clara, CA, USA) was equipped with a circulated heated/chilled water bath (VWR 1150A, VWR, Radnor, PA, USA). The Cys stock solution (206 mM) was prepared by dissolving 12.5 mg of L-cysteine in 500 mL phosphate buffer (0.1 M, pH 5.6). The Cys-alkylated tuna MbFe(III)-H_2_O-NEM and horse MbFe(III)-H_2_O (hMbFe(III)-H_2_O) were used as controls. It is pertinent to recall that horse Mb has no Cys residue in its amino acid sequence (UniProt no. P68082).

### Spectral characterization of tuna ferrylmyoglobin (MbFe(IV)=O) and tuna sulfmyoglobin (MbFe(II)-S)

To determine whether the intermediate product during thermal treatment of tuna MbFe(III)-H_2_O in the presence of Cys was MbFe(IV)=O, we experimentally produced tuna MbFe(IV)=O using the method reported by Carlsen et al. (2000). The tuna MbFe(III)-H_2_O solution (0.2 mg/mL) in phosphate buffer (0.1 M, pH 5.6) was reacted with H_2_O_2_ in a 1:3 molar ratio. The production of tuna MbFe(IV)=O was monitored using a UV-vis Cary 50 at room temperature, monitoring spectral changes every 15 s for 225 s reaction.

To compare the spectra of tuna MbFe(II)-S produced by thermal treatment in the presence of Cys, production of tuna MbFe(II)-S from tuna MbFe(IV)=O reduction by Cys at room temperature was conducted following the method reported by Libardi et al. (2014) with modifications. First, the tuna MbFe(IV)=O (0.2 mg/mL), previously produced, was reduced with Cys (4 mM) at room temperature, and the spectra were recorded using a UV-vis Cary 50 spectrophotometer (Varian, Inc. Agilent Technologies, Santa Clara, CA, USA). Finally, to induce the MbFe(II)-S oxidation state of desoxysulfmyoglobin, the system was slightly heated to 40 °C, and its absorbance spectra were monitored as before.

### Kinetics of tuna sulfmyoglobin (MbFe(II)-S) production

Due to the only involvement of Cys in the GR, the kinetic study was conducted by varying Cys concentration (at 1, 2, 4, and 6 mM), making sure to have an excess of establishing pseudo-first-order conditions since the Cys concentration in Bigeye tuna (*Thunnus obesus*) muscle is 0.04 mM (Yi & Xie, 2022) and monitoring the MbFe(II)-S formation at 610 nm during the thermal treatment. The pseudo-first-order rate constants were obtained by fitting the experimental data to a first-order equation using Origin Pro 2018. Moreover, to evaluate the involvement of H_2_O_2_ in the reaction, catalase (25–100 U) was incorporated into the 4 mM Cys system.

## Results

### Purification of tuna Mb

**Figure 1:**
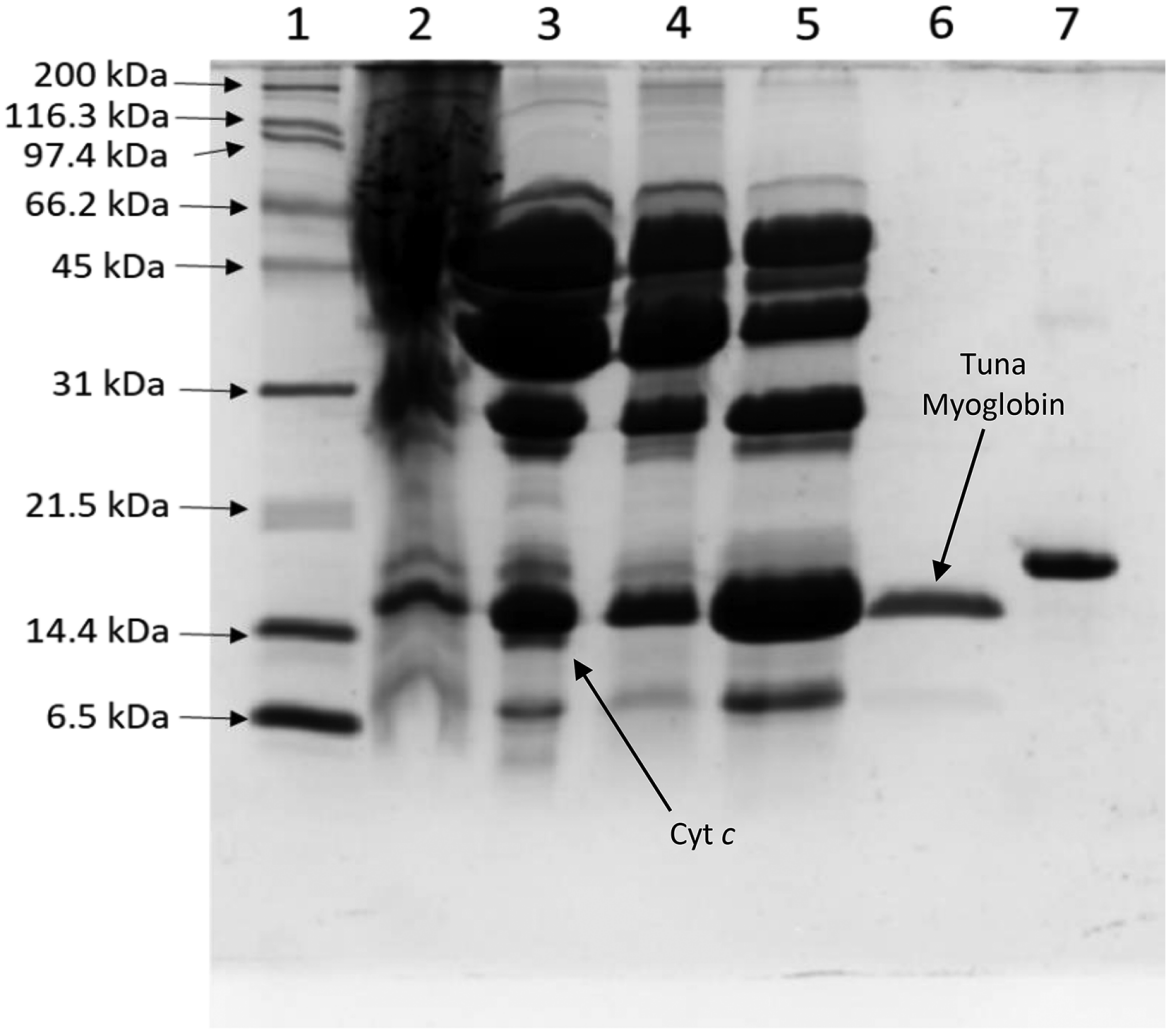
Process of tuna myoglobin purification monitored by SDS-PAGE (17%). The lanes represent: (1) molecular weight marker, (2) crude extract, (3) 65–100% ammonium sulfate saturation fraction, (4) SP HP fraction, (5) sephacryl S-200 fraction, (6) purified tuna myoglobin, (7) horse myoglobin. Cyt *c*: Cytochrome *c*.

**Figure 2:**
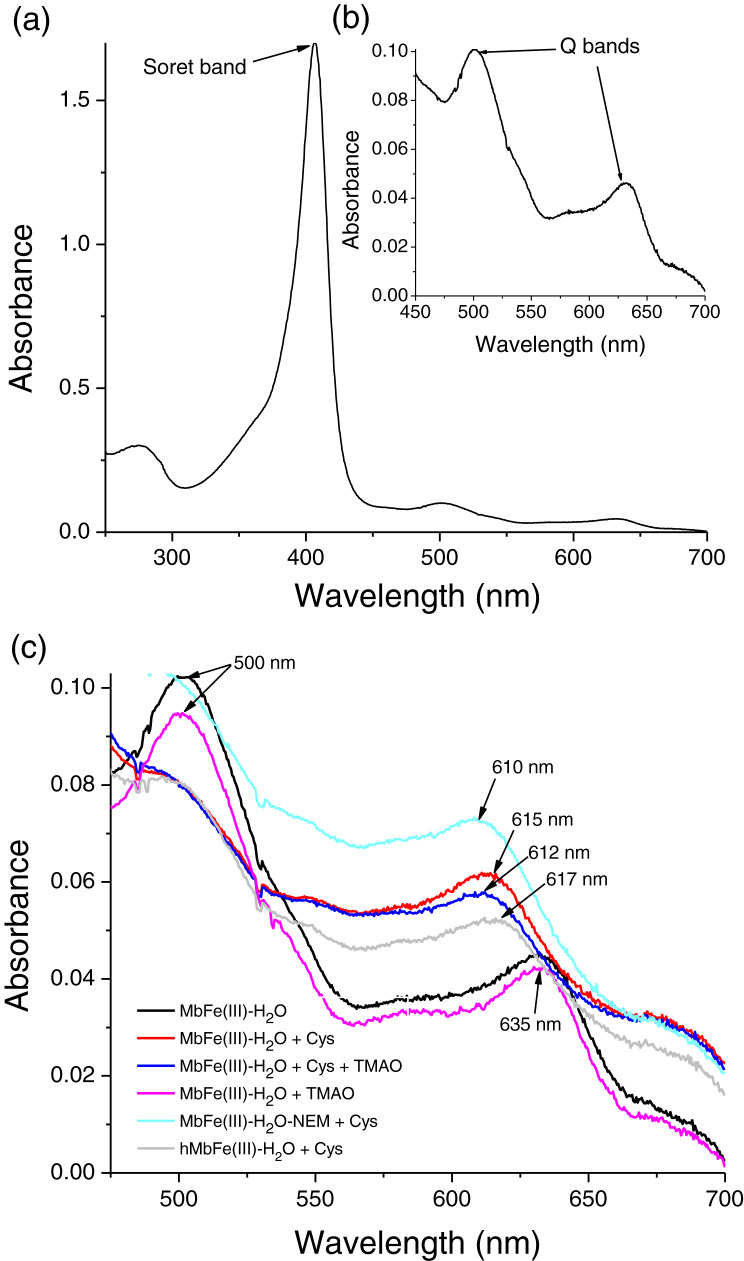
Spectral characterization (at 250–700 nm) of tuna metmyoglobin (MbFe(III)-H_2_O). (A) Soret band, (B) Q bands and (C) Q region spectrum of tuna MbFe(III)-H_2_O after thermal treatment (60 °C/30 min). Treatments: only MbFe(III)-H_2_O (Black), MbFe(III)-H_2_O + Cys (Red), MbFe(III)-H_2_O + Cys + TMAO (Blue), MbFe(III)-H_2_O + TMAO (Pink), MbFe(III)-H_2_O-NEM+Cys (Green) and hMbFe(III)-H_2_O (Horse metmyoglobin) + Cys (Gray). MbFe(II)-S: Sulfmyoglobin.

A myoglobin purification protocol was implemented to eliminate any possible cytochrome *c* contamination. SDS-PAGE monitored the tuna Mb purification protocol (Fig. 1), and it showed purity to assess further spectroscopic characterization (Fig. 1, lane 6) as described above. The UV-vis absorption scan confirmed the purity of tuna Mb showing a heme-absorption Soret band (407 nm) (Fig. 2A) and two Q bands (at 500 and 635 nm) (Fig. 2B) characteristics of Mb (Thiansilakul, Benjakul & Richards, 2011).

### Spectral changes of tuna metmyoglobin (MbFe(III)-H_2_O), free Cys, and/or TMAO solutions during thermal treatment

**Figure 3:**
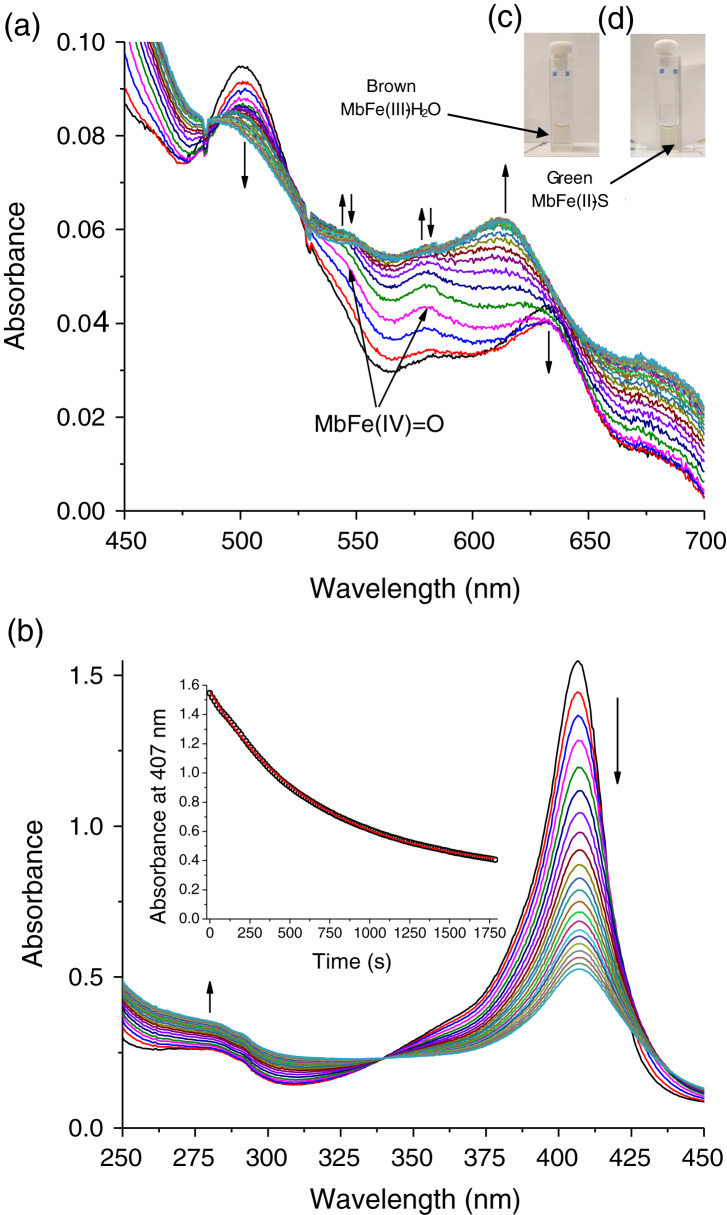
Production of tuna sulfmyoglobin (MbFe(II)-S) in the presence of 4 mM free cysteine (Cys) during thermal treatment (60 °C/30 min). (A) Spectral changes in the Q region. (B) Spectral changes in the absorbance at 280 nm and Soret band. Readings every minute. Inset represents the changes of Soret band (407 nm) absorbance with respect to time. The vertical arrows changes in absorbance with respect to time indicate Tuna ferrylmyoglobin: MbFe(IV)=O.

The tuna greening process is triggered by heat. However, the temperature used during heating in this work was 60 °C since higher values caused protein aggregation, making it impossible to monitor the spectral changes during heating. In our work, the tuna Mb had spectroscopic changes during the reaction with Cys and TMAO at 60 °C. Visually, the solution turned from brown to green (Figs. 3C and 3D, respectively), leading to a reduction in the intensity of the Soret band (407 nm) (Fig. 3B) and Q bands (503 and 630 nm) (Fig. 3A) corresponding to MbFe(III)-H_2_O with a reddish hue. The green color wavelength (~610 nm) increases (Fig. 3A).

The main spectral change of MbFe(III)-H_2_O during the Cys and TMAO reaction is the reduction of the Soret band intensity (Fig. 3B). This process was kinetically monitored with a constant of 1.47 × 10^−3^ s^−1^ (inset in Fig. 3B).

In contrast, thermal treatment of MbFe(III)-H_2_O with Cys plus TMAO (Fig. 2C) showed a similar spectrum when compared with the treatment in the absence of TMAO (only Cys), both showing the formation of sulfmyoglobin (MbFe(II)-S) (band at 615 nm), being evident that only Cys promotes the GR. Besides, the treatment with only TMAO and MbFe(III)-H_2_O (Fig. 2C) showed a similar spectrum to that of the control (only metmyoglobin, MbFe(III)-H_2_O) after thermal treatment with no change; thus, a discoloration reaction did not occur by thermal treatment in the presence of only TMAO.

### Cys-10 in the greening reaction

In this work, the treatment consisting of MbFe(III)-H_2_O with its Cys-10 blocked (MbFe(III)-H_2_O-NEM) and Cys also produced MbFe(II)-S (band at 610 nm) after thermal treatment (Fig. 2C). It is essential to mention that horse metmyoglobin (hMbFe(III)-H_2_O) was employed as a control since this Mb does not have cysteines in its primary structure; however, the GR also developed in the presence of Cys (Fig. 2C).

### Ferrylmyoglobin (MbFe(IV)=O) as an intermediate in the greening reaction

The spectral changes of MbFe(III)-H_2_O in the presence of Cys during thermal treatment show MbFe(II)-S formation. Still, the formation of a reaction intermediary is precise (Fig. 3A). The intermediary spectrum has two bands, at 545 and 580 nm, characteristics of ferrylmyoglobin (MbFe(IV)=O).

**Figure 4:**
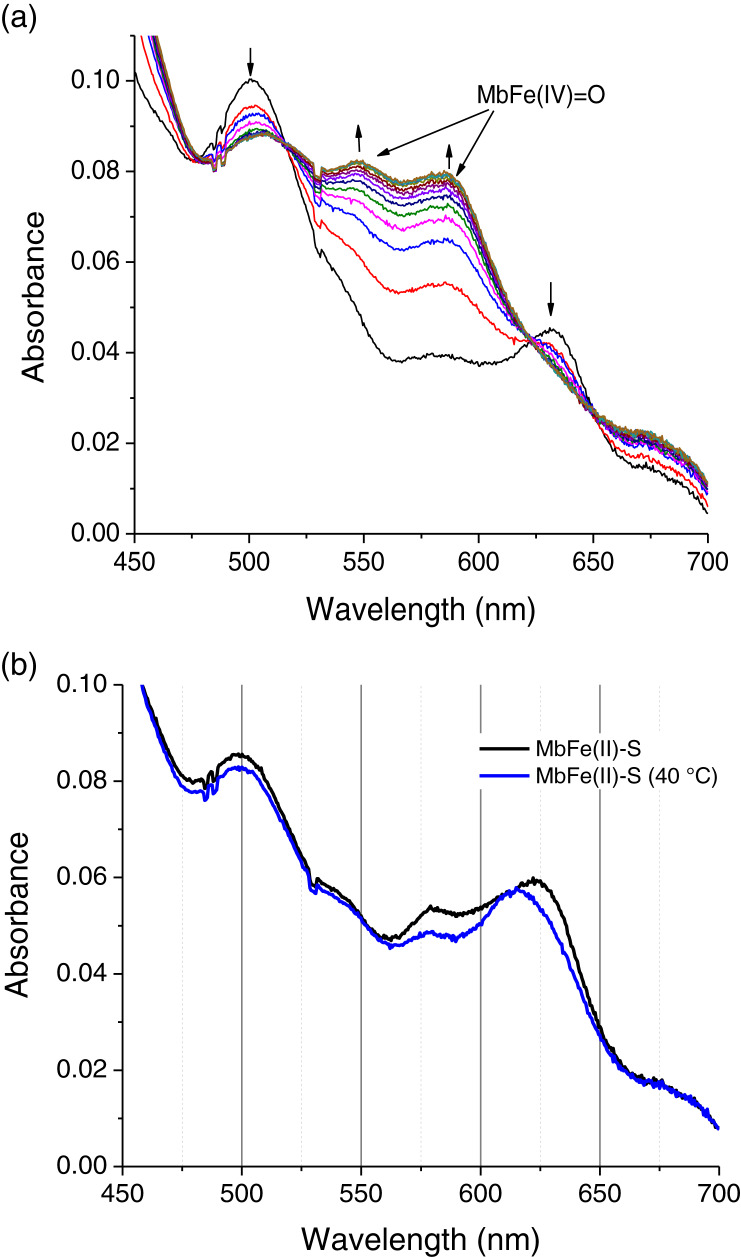
Production of tuna ferrylmyoglobin (MbFe(IV)=O) and Sulfmyoglobin by interaction with H_2_O_2_ and cysteine (Cys), respectively. (A) Production of tuna ferrylmyoglobin (MbFe(IV)=O) at room temperature. Readings every 15 s. The up and down arrows indicate formation of MbFe(IV)=O and consumption of tuna metmyoglobin (MbFe(III)-H_2_O), respectively. (B) Spectra of tuna sulfmyoglobin (MbFe(II)-S) produced by reduction MbFe(IV)=O in the presence of Cys (4 mM) at room temperature (black line) and at 40 °C (blue line).

To confirm the MbFe(IV)=O as a reaction intermediary, this compound was produced experimentally by a reaction between tuna MbFe(III)-H_2_O and H_2_O_2_ (Carlsen et al., 2000). Figure 4A shows the process of production of MbFe(IV)=O from tuna MbFe(III)-H_2_O, and as it is observed, the MbFe(III)-H_2_O spectra increase in bands around 545 and 580 nm (Carlsen et al., 2000; Romero et al., 1992). Moreover, the tuna MbFe(II)-S was also experimentally produced by reaction with Cys and H_2_O_2_ at room temperature (Fig. 4B), confirming that the reaction intermediary of the GR is MbFe(IV)=O. One interesting tuna MbFe(II)-S feature was the blue-shift of the main absorption band from 622 to 610 nm after heating at 40 °C (Fig. 4B). Besides, the use of catalase reduced MbFe(II)-S production (see next section).

### Kinetics of tuna MbFe(II)-S production

**Figure 5:**
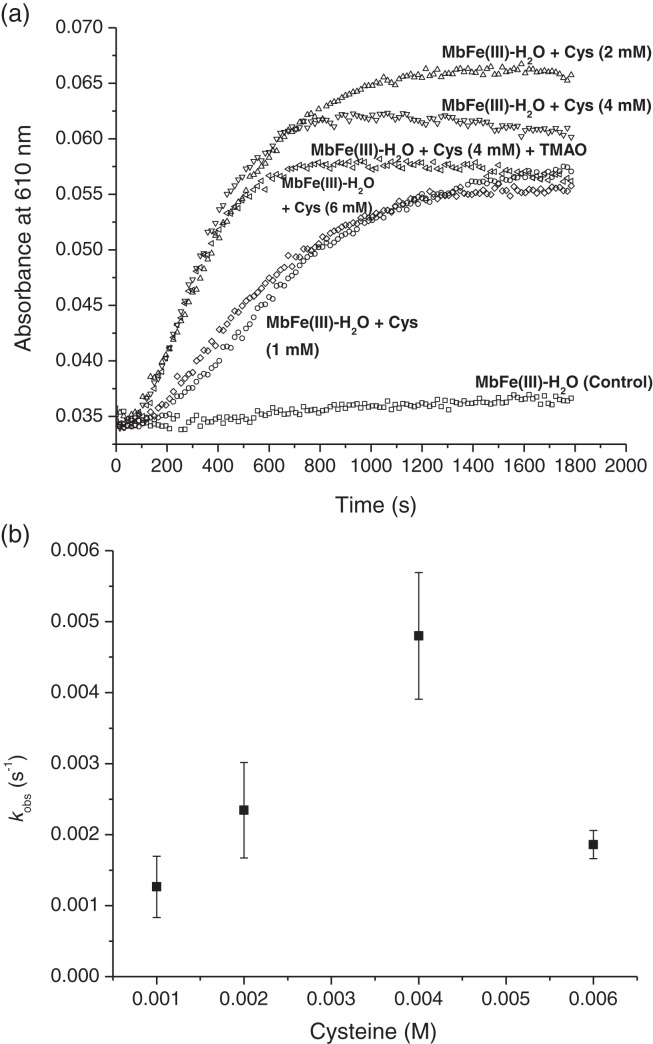
Kinetics of tuna greening reaction promote by Cys during thermal treatment. (A) Tuna sulfmyoglobin (MbFe(II)-S) production through time from the thermal treatment (60 °C, pH 5.6) of tuna metmyoglobin (MbFe(III)-H_2_O) at different cysteine (Cys) concentrations. (B) Rate profile for the formation of tuna sulfmyoglobin (MbFe(II)-S) with respect to the Cys concentration during thermal treatment (60 °C).

The production of tuna MbFe(II)-S by the reaction between tuna MbFe(III)-H_2_O and Cys was followed at 610 nm through time (Fig. 5A). In all experiments, MbFe(II)-S generation exhibited pseudo-first-order kinetics behavior, indicating a dependence of the observed rate constant (*k*_obs_) on the Cys concentration. The Cys concentration profile (Fig. 5A) shows maximum absorbance at 2 mM of Cys (the time for the production of MbFe(II)-S being 1,000 s); meanwhile, when the Cys concentration was 1 and 6 mM, the GR took longer (around 1,400 s). Moreover, the results showed that the addition of 1.33 mM TMAO to the MbFe(III)-H_2_O + Cys treatment decreased MbFe(II)-S production (Fig. 5A).

To obtain the observed rate constants for each Cys concentration (Fig. 5B), Eq. (1) was used, and all kinetics profiles were fitted by exponential analysis. A maximum dependence on the Cys concentration was observed at 4 mM with *k*_obs_ of 4.80 ± 0.89 × 10^−3^ s^−1^. The incorporation of 1.33 mM TMAO into this treatment did not influence its *k*_obs_ (5.17 ± 0.14 × 10^−3^ s^−1^, data not plotted). Nevertheless, the MbFe(II)-S production and reaction rate at 6 mM Cys (1.86 ± 0.20 × 10^−3^ s^−1^) was considerably reduced and comparable to the 1 mM Cys (1.26 ± 0.43 × 10^−3^ s^−1^) (Figs. 5A and 5B).

(1) $${A_{\left( t \right)}} = \; {A_o} + {A_{inf}}\left( {1 - {e^{ - kt}}} \right)$$

where *A*_*(t)*_ is absorbance through time, *A*_*0*_ is initial absorbance, *A*_*inf*_ is final absorbance, and *k* is the rate constant.

**Figure 6:**
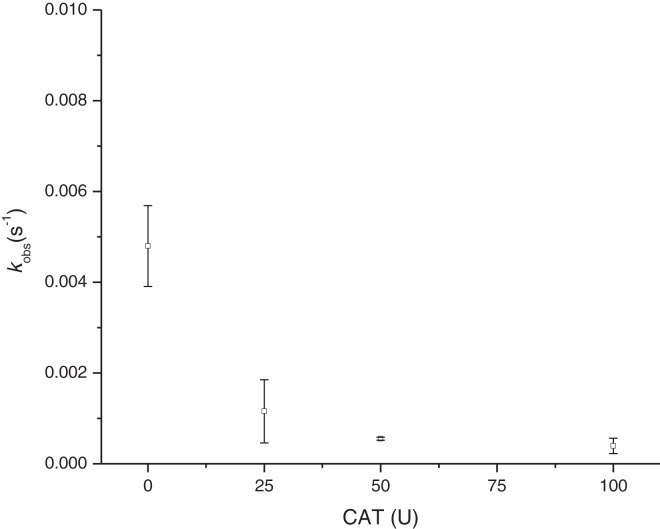
Effect of catalase in the rate constant of sulfmyoglobin formation at pH 5.6 and 4 mM Cys. CAT, catalase from the bovine liver; U, units of enzyme activity; *k*_obs_, observed rate constant.

On the other hand, the incorporation of catalase into the reaction (at 4 mM Cys) (Fig. 6) showed the dependence of H_2_O_2_ in the MbFe(II)-S formation, as its *k*_obs_ were reduced and practically inhibited when catalase activity was increased (*i.e*., *k*_obs_ of MbFe(II)-S formation was reduced to 1.16 ± 0.69 × 10^−3^ s^−1^ at 25 U of catalase used).

## Discussion

### Spectral changes of purified tuna metmyoglobin (MbFe(III)-H_2_O) during thermal treatment with Cys and/or TMAO

The Mb purification protocol helped to remove the cytochrome *c* hemeprotein, which has a similar molecular weight (11.5 kDa) (Tanaka et al., 1975) compared to Mb (15.4 kDa) (Ueki & Ochiai, 2006).

The changes on the Soret band (407 nm) during thermal treatment (60 °C/30 min) indicate conformational changes in the heme cavity without the heme group dissociation (Cheng et al., 2018; Kundu et al., 2015) that it is associated only with GR (inset Fig. 3B).

**Scheme 1:**
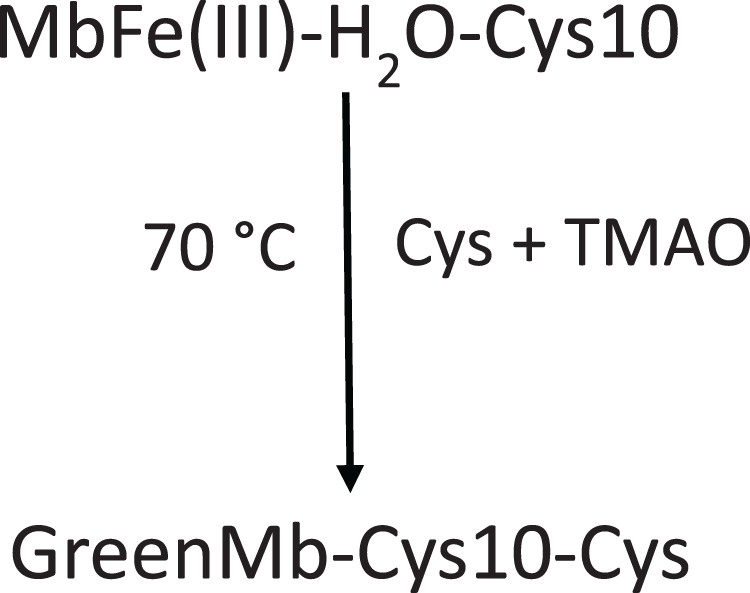
Scheme proposed by Grosjean et al. (1969) for tuna greening after thermal treatment (70 °C) by interaction among tuna metmyoglobin (MbFe(III)-H_2_O), trimethylamine oxide (TMAO) and free cysteine (Cys). Cys-10 represents the conserved cysteine residue in tuna myoglobin.

Grosjean et al. (1969) stated that Cys promoted MbFe(III)-H_2_O greening after thermal treatment (70 °C) and under the presence of TMAO that catalyzed the disulfide bonds between Cys-10 and Cys (Scheme 1). The increase in the green color wavelength (610 nm) is due to the MbFe(II)-S production in a desoxy state (Fig. 3A) (Libardi et al., 2014; Berzofsky, Peisach & Blumberg, 1971), indicating that tuna GR is related to MbFe(II)-S and not to cholemyoglobin (628 nm) and verdomyoglobin (760 nm) (Yong et al., 2018). Although this has been thoroughly described in the literature, the chemical basis for the GR process in tuna muscle remains in discussion.

In our study, we show that TMAO did not have any effect on the GR (Fig. 2C), a result that is not consistent with Grosjean et al. (1969) and Koizumi & Hashimoto (1965), who mentioned that the presence of TMAO is necessary to induce the tuna GR.

Thermal activation of the GR has been described, which does not occur at room temperature (25 °C) and physiological pH (Álvarez et al., 2020; Libardi et al., 2014). Also, gamma radiation in the presence of Cys promotes the MbFe(II)-S production (Motohashi et al., 1981). In this respect, the formation of MbFe(II)-S by Cys has not been reported since it is well known that MbFe(II)-S production is related to the Mb-H_2_S or H_2_O_2_ interaction (Faustman & Suman, 2017; Libardi et al., 2013). Hence, tuna MbFe(II)-S production is due to a Cys oxidation process during thermal treatment.

### Cys-10 in the greening reaction

The Cys-10 is a conserved amino acid in tuna myoglobins that has been identified as one of the factors responsible for tuna greening (Grosjean et al., 1969; Ueki & Ochiai, 2005). However, in our study of blocking Cys-10, the result confirms that this conserved amino acid present in tuna Mb, which was thought to be a determinant for the GR by thermal treatment and thus exclusively in tunas, does not participate. Besides, the GR also took place on horse MbFe(III)-H_2_O, which lacks this Cys-10.

### Ferrylmyoglobin (MbFe(IV)=O) intermediate in the greening reaction

Ferrylmyoglobin (MbFe(IV)=O) is a hyper-valent state of Mb related to the lipid oxidation in the muscle (Romero et al., 1992; Libardi et al., 2014). From our results, it is evident that the presence of only Cys during thermal treatment (at 60 °C) promotes the oxidation of MbFe(III)-H_2_O to MbFe(IV)=O. In this sense, this reaction was not observed by Pindstrup et al. (2013) and Libardi et al. (2014), who proposed that the reaction between MbFe(III)-H_2_O and Cys could promote the production of MbFe(II)-S at 25 °C and pH 6–8 (without MbFe(IV)=O as an intermediate). Although, in a homologous system like hemoglobin, the reaction with Cys led to the production of the ferrylhemoglobin intermediate (Lips et al., 1996).

**Scheme 2:**
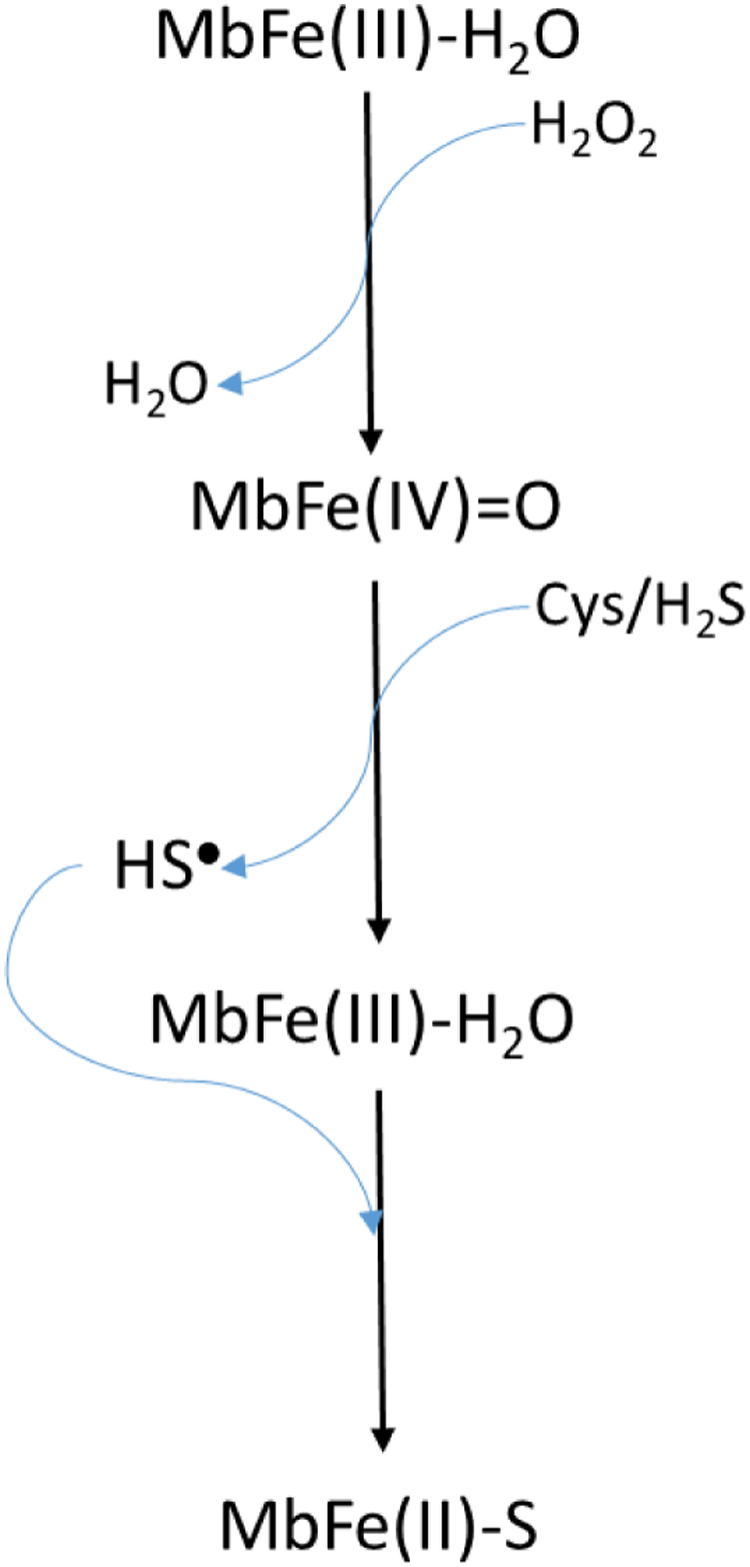
Scheme proposed by Libardi et al. (2013, 2014) for sulfmyoglobin (MbFe(II)-S) production from the reaction among metmyoglobin (MbFe(III)-H_2_O), H_2_O_2_ and thiols componds (Cys/H_2_S).

**Scheme 3:**
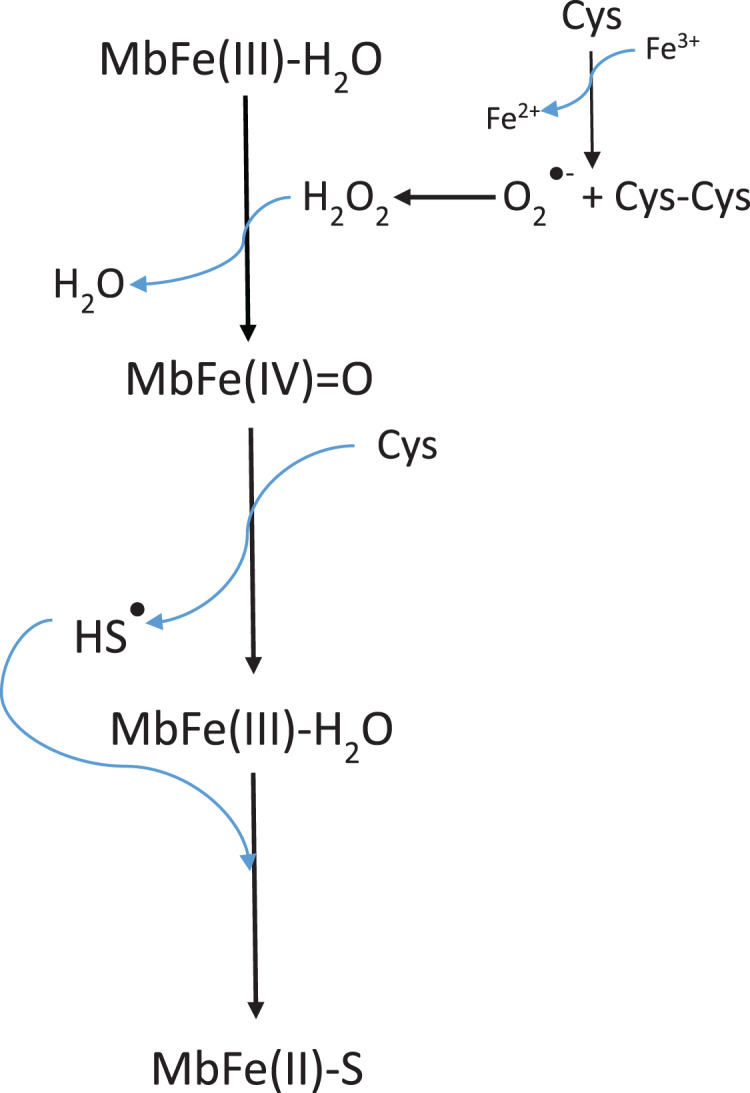
Scheme suggested for the sulfmyoglobin (MbFe(II)-S) production from metmyogloin (MbFe(III)-H_2_O) promoted by free cysteine (Cys) during thermal treatment at 60 °C. The reaction representing the hydrogen peroxide production by free cysteine (Cys) in presence of iron (Fe^3+^) was proposed by Padovani et al. (2016).

Libardi et al. (2013) model (Scheme 2) requires the presence of H_2_O_2_ to produce MbFe(IV)=O from MbFe(III)-H_2_O, and subsequently, the MbFe(IV)=O reduction to MbFe(III)-H_2_O by Cys forming sulfhydryl radicals (HS^•^) which finally react with the MbFe(III)-H_2_O heme group comprising MbFe(II)-S (Libardi et al., 2014; Pindstrup et al., 2013; Romero et al., 1992). However, we propose (Scheme 3) that H_2_O_2_ is produced *via* Cys oxidation, promoted by the prooxidant activity and exposition of heme iron from MbFe(III)-H_2_O during heating (Kristensen & Andersen, 1997) since the incorporation of catalase into the reaction system, affected the kinetics of the GR due to the decrease of H_2_O_2_ available for the formation of MbFe(IV)=O (Giulivi & Cadenas, 1993).

**Scheme 4:**
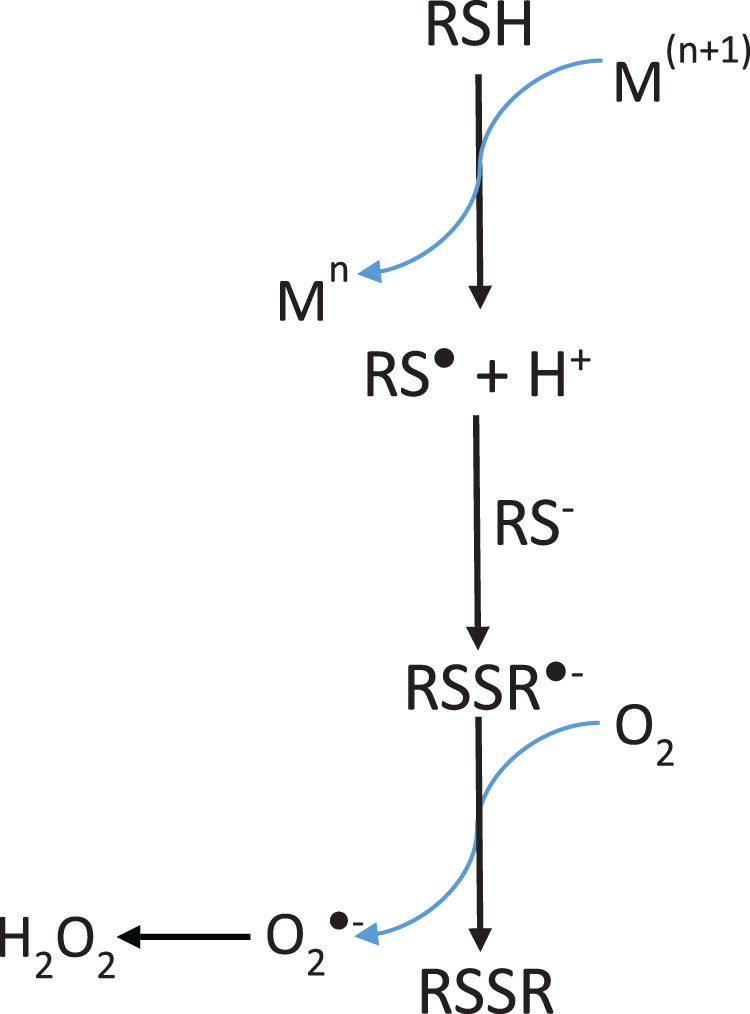
Mechanism of hydrogen peroxide formation by thiol-containing compounds (RSH) oxidation induced by transition metals (M). Adapted from Padovani et al. (2016).

It is evident that Cys during thermal treatment promotes the MbFe(III)-H_2_O oxidation to MbFe(IV)=O and the subsequent production of MbFe(II)-S. In this sense, it has been established that the oxidation mechanism of Cys in the presence of metals (such as iron and copper) produces superoxide anion (O_2_^•−^), H_2_O_2_ (by dismutation of O_2_^•−^), and thiyl radicals (RS^•^) (Scheme 4) (Padovani et al., 2016). Being H_2_O_2_ and RS^•^ the oxidation products involved in the formation of MbFe(IV)=O and MbFe(II)-S (Scheme 3), respectively, their production is promoted by iron release from MbFe(III)-H_2_O during thermal treatment (Purchas et al., 2003). Besides, it has been reported that the reaction of Cys with heme proteins generates O_2_^•−^ and H_2_O_2_ and that some experimental conditions (as those used in our study) could facilitate the release of iron, favoring pro-oxidant conditions (Romero et al., 1992; Soladoye et al., 2015).

The tuna MbFe(II)-S blue-shift is probably due to the reduction in oxygen concentration produced by the heat treatment (40 °C) (Fig. 4B), which might affect this compound’s oxy/redox state. This difference in MbFe(II)-S spectra is possibly due to the oxidation state of MbFe(II)-S found in model systems; in this sense, Berzofsky, Peisach & Blumberg (1971) determined that the oxy sulfmyoglobin and desoxy sulfmyoglobin have different spectra with a maximum peak at 623 and 616 nm, respectively.

### Kinetics of tuna MbFe(II)-S production and effect of catalase

The kinetics results of the present study confirmed that TMAO does not promote an increase in the GR through MbFe(II)-S production. Besides, Libardi et al. (2014) report that the increment in the *k*_*obs*_ from the MbFe(IV)=O + Cys reaction is Cys concentration-dependent. Nevertheless, we have shown that such dependence is limited to a 1–4 mM Cys concentration range starting from a MbFe(III)-H_2_O + Cys system. However, the *k*_*obs*_ of MbFe(II)-S production is reduced at 6 mM Cys since high Cys concentrations (with respect to protein) promote structural protein perturbation during thermal treatment (Boye, Ma & Ismail, 2004).

Therefore, our results suggest that for the GR, by MbFe(II)-S production of tuna MbFe(III)-H_2_O, to take place at postmortem pH, the thermal treatment and only the presence of Cys are needed.

On the other hand, the GR is promoted by H_2_O_2_ production since the incorporation of catalase reduces the *k*_obs_ of MbFe(II)-S production (Giulivi & Cadenas, 1993). This result is consistent with the proposed mechanism (Scheme 3), showing that the GR is a consecutive reaction with MbFe(IV)=O as an intermediary compound.

## Conclusions

The greening reaction of skipjack tuna (*Katsuwonus pelamis*) muscle is related to MbFe(II)-S production through the reaction between MbFe(III)-H_2_O and Cys, promoted by the generation of oxidation products of Cys, such as H_2_O_2_, O_2_^•−^ and thiyl radicals during heating. Furthermore, although TMAO is a common compound in tuna muscle, it does not intervene in muscle greening. It is essential to mention that the greening is a consecutive reaction, with MbFe(IV)=O as an intermediary. The greening reaction has also been classified as a discoloration exclusively of tuna species due to the presence of Cys-10. In this regard, it is concluded that Cys-10 is not essential for the greening reaction since this reaction can also be developed in other muscle systems.

## Supplemental Information

10.7717/peerj.13923/supp-1 Supplemental Information 1 Raw data of scans, kinetics and image of electrophoresis gel. The raw data in .opj format require Origin software to view. The image of electrophoresis gel is present in .scn format, it is necessary to use the software Image Lab to view.

## Additional Information and Declarations

### Data Availability

The following information was supplied regarding data availability: All raw scans and kinetics are available in the Supplemental File.
